# The Experiences of Close Relatives to Women with Chronic Obstructive Pulmonary Disease Stages III or IV: A Qualitative Study

**DOI:** 10.3390/nursrep13030086

**Published:** 2023-07-15

**Authors:** Ann Ekdahl, Siv Söderberg, Malin Holmström Rising

**Affiliations:** Department of Health Sciences, Mid Sweden University, SE-852 33 Sundsvall, Sweden

**Keywords:** chronic illness, close relatives, chronic obstructive pulmonary disease, interviews, qualitative content analysis, support

## Abstract

Chronic obstructive pulmonary disease stage III or IV is a progressive and incurable disease. The hallmark of the disease is breathlessness, and it is graded into four different stages, from mild to severe. Living with chronic obstructive pulmonary disease impacts almost every aspect of everyday life for an affected person. As the illness progresses to stages III and IV, the need for support from close relatives increases. The aim of this study was to explore and describe the experiences of close relatives of women with chronic obstructive pulmonary disease stage III or IV and it used qualitative content analysis of individual, semi-structured interviews. Close relatives (n = 9) were interviewed about their experience of being close to a woman with chronic obstructive pulmonary disease stage III or IV. They experienced stress and uncertainty in an unpredictable everyday life. Close relatives supported the women both practically and emotionally and they called for tailored information about the illness, considering it as an essential tool for support. The results highlighted that healthy close relatives had difficulty in understanding the experience of living with chronic obstructive pulmonary, as they take the simple fact of breathing for granted most of the time.

## 1. Introduction

Chronic obstructive pulmonary disease (COPD) is a progressive and incurable disease, diagnosed through the presence of persistent airflow limitation and graded in four different stages, from mild to severe. The global prevalence of COPD is 11.7% and people living with COPD are mainly over 40 years of age [1]. The hallmarks of COPD are breathlessness, fatigue, cough and sputum production [2,3]. It is common for the disease course to proceed with periods of acute lung exacerbation and periods of recovery and less acute symptoms [4]. COPD is associated with a high burden of comorbidity. A key environmental risk factor for COPD is cigarette smoking [1].

Living with COPD has a significant impact on almost every aspect of everyday life for the ill person due to breathlessness and additional symptom burden [1,5,6,7,8,9,10]. Close relatives are very important persons for people with long-term illnesses such as COPD [11,12]. COPD stage III or IV is an unpredictable illness associated with uncertain outcomes, which means that people living with COPD need help and support from close relatives to manage everyday life [13,14,15]. As the illness progresses to stages III–IV, the need for support increases from close relatives [1,16,17]. Research has shown that support from close relatives is salient for women with COPD stage III or IV to strengthen their ability to manage daily life [7]. Close relatives of people with COPD stage III or IV are emotionally burdened, as people with severe COPD have an exceptionally unpredictable illness, causing acute exacerbation and fear of breathlessness [11,12,13]. This means that support required from close relatives changes over time and their daily life becomes increasingly influenced by the course of COPD development in the affected individual [11,16].

To the best of our knowledge, there is no previous study describing the experience of close relatives of women with COPD stage III or IV. Understanding this experience could help to meet the needs of close relatives and guide healthcare personnel to give tailored support that meets their needs and resources. Thus, this study aims to explore and describe the experiences of close relatives of women with COPD stage III or IV.

## 2. Materials and Methods

### 2.1. Design

A qualitative methodological and exploratory design was used in this study, to achieve the aim of describing the experience of close relatives to women with COPD stage III or IV. Semi-structured interviews were conducted with close relatives and the interview texts were analysed using qualitative content analysis according to Graneheim and Lundman [18]. COPD stage III or IV will herein be referred to as COPD to facilitate the readability of the paper. Consolidated criteria for reporting qualitative research were used (QOREQ File S1) [19].

### 2.2. Context

The study was performed in a region of central Sweden. This region has a population of 245,000 inhabitants and contains both rural and urban areas [20]. In Sweden, the estimated prevalence of all stages of COPD is between 500,000 and 700,000 people (approximately 6 percent), with the prevalence of stages III and IV being approximately 49,000 people [2,21].

### 2.3. Participants and Procedure

A sample of nine close relatives participated in this study. Women with COPD who participated in a previous study [7] invited a person (persons the women identified as close relatives) to participate in an interview, and provided information and informed consent. Once the consent form was returned by a close relative, interviews were arranged at a time convenient for those close relatives who agreed to participate. The participants were five women and four men, with ages ranging from 45 to 85 years (median = 57 years). Six had completed secondary school and three had obtained higher education. Five were working, while the other four were retired. Four of the participants lived with a woman with COPD. None of the close relatives in this study was an informal caregiver for someone with COPD.

### 2.4. Interviews

Individual, semi-structured interviews were conducted with the participants, based on questions about their experience as close relatives of a woman with COPD [22]. The interviews were conducted via phone, following the recommendations for social distancing due to COVID-19. An interview guide (Table 1) with a focus on close relatives’ everyday life was used. Follow-up questions were asked, such as ‘Can you tell me more about that?’ and ‘Can you give an example?’ The interviews were conducted from March to May 2020, and were digitally recorded and later transcribed verbatim. The interviews lasted between 39 and 76 min (median = 62 min).

### 2.5. Data Analysis

Qualitative content analysis following Graneheim and Lundman [18] was used to analyse the interview texts. The analysis was performed stepwise. Firstly, the interview texts were read several times to get a sense of the content. Secondly, guided by the aim of the study, meaning units were identified, condensed and labelled with codes. Thirdly, coded meaning units were then sorted into categories based on similarities and differences. Lastly, the categories were subsumed into four themes. Table 2 presents an example from the analysis process.

### 2.6. Ethical Considerations

All participants gave their informed consent to participate in this study. They received oral and written information regarding the study before the interviews and were informed that their participation was voluntary and that they could withdraw at any time without explanation. They were guaranteed confidentiality and anonymity in the presentation of the findings. Interview texts were accessible only by the authors. The study was conducted in accordance with the Declaration of Helsinki [23] and was approved by the Ethical Review Agency in Sweden (Dnr: 2020-00085).

## 3. Results

The results are grouped into the following four themes: difficulty in understanding the woman’s ill health; experience of stress and uncertainty related to the woman’s ill health; giving practical and emotional support; lack of tailored information about COPD. These themes are illustrated with referenced citations from the interviews with close relatives (CR 1–9).

### 3.1. Difficulty in Understanding the Woman’s Ill Health

Close relatives reported that they had become used to changes and a gradual deterioration in the woman’s ill health. Despite the ever-present breathlessness, coughing and various treatments, they emphasised that it was hard for them to understand the woman’s situation caused by COPD. They expressed the need to get help to better understand the woman’s ill health. Close relatives expressed that intimacy was affected by the woman’s limited energy:

“I did not realize how badly the illness affects her …. It just gets worse and worse, and I did not understand it….”

(CR7)

Close relatives expressed that they remembered when the women were healthy and had the energy to manage everyday life, and when the women had the opportunity to socialise spontaneously and participate in sports activities.

“it is important to create new opportunities together and share difficulties….”

(CR8)

It was also difficult for close relatives to fully understand the consequences of the women’s lack of energy. They expressed that it was impossible to continue everyday life as before and therefore their mutual activities had changed. Close relatives were frustrated when attempting to continue these activities and underlined the importance of creating new opportunities to socialise and share difficulties in everyday life together with the women.

### 3.2. Giving Practical and Emotional Support

Close relatives described the importance of giving practical and emotional support in various ways in everyday life to the women, such as buying groceries, providing transport to medical appointments and helping with housework. The COVID-19 pandemic forced close relatives to increase their awareness of how they provided support to avoid infecting the women, and social distancing was already a well-established strategy for close relatives of those with COPD. To maintain the women’s functionality in everyday life, close relatives supported them to be active:

“I feel good about helping her [the woman] … and I feel good if she gets better from my help.”

(CR2)

Close relatives gave emotional support via phone, text, or Skype. Social media enabled close relatives to communicate more easily to keep in touch with the woman with COPD despite physical separation. They expressed that their support sometimes affected their own everyday life and rhythm:

“I must be available… my everyday life is affected extensively.”

(CR6)

Close relatives wanted to be supportive during working hours, particularly following the outbreak of the COVID-19 pandemic. They described stretching themselves, sometimes to breaking point:

“I am on standby.... I call her every morning and I talk to her after work because I understand that if she gets ill during the day, especially now, if it’s COVID-19, it can take three hours and then she’ll be dead.”

(CR4)

### 3.3. Experiencing Stress and Uncertainty Related to a Woman’s Ill Health

Close relatives perceived everyday life as stressful and uncertain. It was worrying for them to experience the progression of the woman’s illness and to know that the illness is uncurable. Due to COPD being a progressive illness, everyday life was an unpredictable situation for the women as well as for close relatives. When the condition worsened, close relatives had to show great consideration. They mentioned situations when they felt grief, anxiety and sadness and cried due to the hopelessness that accompanies the illness.

“…the days that are worse [for the woman]...you must tiptoe around and be careful of words and actions….”

(CR3)

“Their panic is painful to endure …. There are many times you cry due to hopelessness …. It’s stressful to see.”

(CR1)

Close relatives felt guilty when they lacked time or had to go away on a work trip or vacation. Feelings of worry for the women with COPD increased after the outbreak of the pandemic. It was challenging for close relatives being unable to reduce the women’s breathlessness, and they found it difficult to know how to give support in this situation.

“… when she [the woman] lived in another city, I had a bad conscience because I could not be present to support her… I couldn´t be available when she [the woman] got hospitalized—those worries for me … then I had a bad conscience.”

(CR5)

### 3.4. Lack of Tailored Information about COPD

Close relatives described how they lacked tailored information about COPD which they thought would be helpful. They called for a platform for the exchange of information and an opportunity for dialogue where they could ask questions about the illness, preferably without the affected women being present. In addition, close relatives wanted the necessary support in terms of information regarding the progress of the illness and its complications. Close relatives expressed having read about COPD involving a ‘fading away’ towards an unpleasant death, and in connection with this, they had initiated sincere end-of-life discussions with the women. Close relatives believed that if they received tailored information, it would increase their ability to support the women with COPD.

“I also thought that there should have been someone special to call for both me and [the woman] to talk to about support even about end-of life.”

(CR9)

## 4. Discussion

This study aimed to explore and describe the experiences of close relatives of women with COPD stages III or IV. The results show that the everyday lives of close relatives were impacted by the illness. Close relatives emphasised that despite living close to the women with COPD, regardless of the type of relationship they had with each other, they had difficulty fully understanding the women’s situation and experienced stress and uncertainty in an unpredictable everyday life. They described how they supported the women both practically and emotionally and highlighted the need for tailored information about COPD for themselves. Tailored information was regarded by close relatives as an essential tool for support, which would enhance their capacity to help the women with COPD.

Everyday life for close relatives in this study was impacted by the women’s illness. According to Bury [24], chronic illness is a disruptive event that changes the normal structure of family life and requires close relatives to mobilise resources to cope with this disruption. Our results show that close relatives experienced difficulties in understanding the women’s ill health, marked by symptoms of breathlessness and coughing. One reason for this might be that breathing is a universal human experience, highly subjective yet generally carried out ‘invisibly’, without awareness. The experience of breathlessness makes the invisible visible [25]. Carel describes breathlessness caused by chronic illness as a feeling of being trapped, which cannot be compared with the experience of healthy people when, for example, running after the bus [26]. Breathing is something that is generally taken for granted and therefore, it can be hard for close relatives, in the context of COPD, to fully comprehend an everyday life constrained by a lack of energy caused by breathlessness. In addition, our results show that close relatives experienced constant stress and uncertainty in everyday life. It can be exhausting to be the close relative of someone with stage III or IV COPD. Ek et al. [10]. Describe how COPD patients and their relatives live with uncertainty up until the time of death [10,27].

Close relatives described that their everyday life was occupied with giving practical and emotional support to women with COPD to relieve symptoms. According to Toombs [28], chronic illness means that the taken-for-granted aspects of daily living are disrupted. This seems to be also valid for the close relatives in this study, as their everyday lives were significantly affected as a result of supporting women with COPD. In addition, close relatives experienced a lack of support for their own needs and were left to their own devices to figure out how to manage the situation. This finding is in line with Wang et al. [29], who state that the needs of family members of seriously ill persons often remain unmet. These unmet needs require further attention from healthcare providers [30].

However, the close relatives in this study conveyed that they lacked tailored information about COPD that would assist them in understanding and making sense of the illness. Making sense of the experience of living with COPD could be communicated through information about how everyday life with such an illness differs from that of life without [31]. According to Spence et al. [32], close relatives want sufficient information from healthcare professionals. Hansen et al. [33] show that information about COPD enhances close relatives’ feelings of security. Wallengren et al. [34] conclude that the information provided to relatives of stroke survivors must consider individual needs rather than follow generic programs. In this study, close relatives highlighted the need for support for their own psychosocial and existential needs. Corbin and Strauss [35] state that it is important that healthy family members receive information about the sick person’s condition so that it can be incorporated into family life. Van der Smissen et al. [36] conclude that information provided to relatives of people with chronic illness needs to be findable, useful and designed to support the ill person. Additionally, relatives emphasized that it was important to receive information about how to manage their situation and obtain information about where to go if they could not cope. In summary, meeting the informational needs of close relatives is an essential tool for support. Providing information based on their needs and resources enables them to strengthen their ability to provide support to people with COPD in turn.

### Methodological Considerations

To establish reliability (i.e., credibility, confirmability, dependability and transferability), this study has used the approach set out by Lincoln and Guba [37,38]. Credibility was supported by clear inclusion criteria and the heterogeneity of participants, which contributes to a richer variation in understanding the experiences under investigation. The sample size of nine close relatives was based on a model of sample size in qualitative selection and information developed by Malterud et al. [39]. According to this model, sample adequacy and data quality are more important than the number of participants [40]. The interviews gave rich descriptions of the experiences of close relatives. A pilot interview was performed to test the interview guide and no reasons were found to make changes. According to Novick [41], a limitation could be the use of telephone interviews rather than face-to-face meetings. However, due to the pandemic, this was an alternative that safely enabled the collection of data following government regulations. To strengthen confirmability, the data collection and the results were presented as clearly and carefully as possible [42]. Confirmability was additionally sought by including the participants’ descriptions in the results [43]. Furthermore, quotations were selected from the interview texts to reinforce the themes [44] and allow the reader to judge the presented results. Dependability was supported through the conduct of the analysis, with the first author conducting the analysis of data and the two other authors independently checking the results. Lastly, all authors discussed the results and reached an agreement. As the researchers were the key instrument of the study, it was critical to clarify pre-understanding and remain open to background, experience and education during our discussions [44]. All authors are registered nurses with experience in caring for people with long-term illnesses and interacting with close relatives. The findings from this study can be transferred to similar situations if they are recontextualized regarding the specificities of other long-term illnesses [38].

## 5. Conclusions

This study highlights close relatives’ need for tailored information about the experiences of women with COPD. Receiving tailored information can facilitate close relatives’ understanding of the consequences of COPD and thereby increase their capacity to understand the experience of those living with the illness. Tailored information also increases close relatives’ options to receive support for themselves and thus enhances their capacity to support those with COPD. This means that both healthcare providers and patient associations need to be aware of the need to tailor information for close relatives and to co-design this information in collaboration with this group. Digital media provides an opportunity for healthcare providers and patient associations to make such information readily available and accessible to close relatives.

## Figures and Tables

**Table 1:** Interview guide for semi-structured interviews with close relatives.

Item No.	Interview Guide
1	Tell me about your experience of everyday life as a close rela-tive of a woman with COPD.
2	Tell me about an ordinary day.
3	Tell me about your experience when the woman’s illness worsens.
4	Tell me if you have somewhere to turn to if you need support.

**Table 2:** Example of meaning unit, condensed meaning unit, code and theme.

Meaning Unit	Condensed Meaning Unit	Code	Theme
I did not realize how badly the illness affects her … It just gets worse and worse, and I did not understand it…(Close Relative 7)	Did not understand her illness	Women’s ill health	Difficulty in understanding the woman’s ill health

## Data Availability

Data from this study are available on request from the corresponding author.

## Supplementary Materials

The following supporting information can be downloaded at: https://www.mdpi.com/article/10.3390/nursrep13030086/s1. QOREQ File S1: (Consolidated criteria for Reporting Qualitative research) checklist.

## Public Involvement Statement

No public involvement in any aspect of this research.

## Guidelines and Standards Statement

QOREQ: (Consolidated criteria for Reporting Qualitative research) checklist.
